# Vitamin D and VDR in cancer cachexia and muscle regeneration

**DOI:** 10.18632/oncotarget.15583

**Published:** 2017-02-21

**Authors:** Andrea Camperi, Fabrizio Pin, Domiziana Costamagna, Fabio Penna, Maria Lopez Menduina, Zaira Aversa, Teresa Zimmers, Roberto Verzaro, Raffaella Fittipaldi, Giuseppina Caretti, Francesco Maria Baccino, Maurizio Muscaritoli, Paola Costelli

**Affiliations:** ^1^ Department of Clinical and Biological Sciences, University of Turin, Turin, Italy; ^2^ Interuniversity Institute of Myology, Italy; ^3^ Department of Physiology, Complutense University of Madrid, Madrid, Spain; ^4^ Department of Clinical Medicine, Sapienza University of Rome, Rome, Italy; ^5^ Indiana University School of Medicine - IUPUI, Indianapolis, IN, USA; ^6^ Department of Surgery, M.G. Vannini Hospital, Rome, Italy; ^7^ Department of Biosciences, University of Milan, Milan, Italy; Translational Cardiomyology Laboratory, Stem Cell Biology and Embryology, Department of Development and Regeneration, University Hospital Gasthuisberg, Leuven, Belgium

**Keywords:** muscle wasting, regeneration, vitamin D receptor, myogenin, circulating vitamin D

## Abstract

Low circulating levels of vitamin D were associated with decreased muscle strength and physical performance. Along this line, the present study was aimed to investigate: i) the therapeutic potential of vitamin D in cancer-induced muscle wasting; ii) the mechanisms by which vitamin D affects muscle phenotype in tumor-bearing animals.

Rats bearing the AH130 hepatoma showed decreased circulating vitamin D compared to control rats, while muscle vitamin D receptor (VDR) mRNA was up-regulated. Both circulating vitamin D and muscle VDR expression increased after vitamin D administration, without exerting appreciable effects on body weight and muscle mass.

The effects of vitamin D on muscle cells were studied in C2C12 myocytes. Vitamin D-treated myoblasts did not differentiate properly, fusing only partially and forming multinucleated structures with aberrant shape and low myosin heavy chain content. Vitamin D treatment resulted in VDR overexpression and myogenin down-regulation. Silencing VDR expression in C2C12 cultures abrogated the inhibition of differentiation exerted by vitamin D treatment.

These data suggest that VDR overexpression in tumor-bearing animals contributes to muscle wasting by impairing muscle regenerative program. In this regard, attention should be paid when considering vitamin D supplementation to patients affected by chronic pathologies where muscle regeneration may be involved.

## INTRODUCTION

Cachexia is a comorbidity of cancer [1] characterized by progressive loss of body weight as well as of skeletal muscle and adipose tissue mass. More than 50% of all cancer patients develop cachexia. This percentage increases up to 86% during the last 2 weeks of life and about 20% of all cancer deaths can be attributable to cachexia. Moreover, cachexia impairs quality of life, as well as tolerance and response to anti-neoplastic treatments (reviewed in [2]).

Anorexia, inflammation and perturbations of hormonal and metabolic homeostasis significantly contribute to the onset and progression of cachexia. In this regard insulin, angiotensin, leptin and myostatin, together with numerous cytokines, including IL6, IL1 and TNFα have been shown to play a role [2, 3].

Skeletal muscle wasting is among the most important cilnical features of cancer cachexia. It mainly derives from the activation of a protein hypercatabolic response that involves different proteolytic systems, in particular the ubiquitin-proteasome-dependent pathway [4] and autophagy However, other mechanisms have also been suggested to contribute to muscle depletion, such as down-regulation of protein synthesis (see [2]) and impaired myogenic response [5, 6].

Vitamin (Vit) D3, the precursor of biologically active VitD, is a pleiotropic hormone synthesized mainly in the skin via a UV-dependent reaction. VitD3 is then transported to the liver where it is hydroxylated at the C-25 position to produce 25(OH)-VitD, the major circulating form of this hormone. In order to become active, 25(OH)-VitD must be hydroxylated in the C-1 position, producing 1,25(OH)-VitD (hereafter referred to as VitD). This enzymatic reaction takes place mainly in the kidney, although other cells/tissues express the *CYP27B1* enzyme required for hydroxylation [7]. VitD circulates in the blood bound to vitamin D-binding proteins, reaching its target tissues to exert endocrine actions. These latter are mediated by the vitamin D receptor (VDR), a member of the nuclear receptor family of transcription factors, which is expressed in different tissues (reviewed in [8]). VDR knock-out mice show the full phenotype of severe vitamin D deficiency, indicating that VDR is the major mediator of VitD action [9]. Upon binding to VitD, VDR forms a heterodimer with the retinoid-X receptor and regulates the expression of genes whose promoters contain specific DNA sequences known as VDR responsive elements (VDRE). A number of coactivators and corepressors are involved in this transcriptional regulation, providing context, tissue and target gene specificity [8]. However, some actions of VitD have been shown to be more immediate than those described above, and have been suggested to depend on engagement of a membrane associated VDR [10].

The classical and best known functions of VitD involve the regulation of calcium and phosphate metabolism, mainly acting on bone tissue, intestine and kidneys [11]. More recently, VitD has been demonstrated to play a role in several physiological and pathological processes, such as immune response and cancer development [8].

VitD deficiency has been shown to cause abnormalities in skeletal muscle, such as reduced actomyosin content, decrease in mitochondrial Ca^2+^ levels, reduced Ca^2+^ uptake in the sarcoplasmic reticulum and low creatine kinase serum levels [12]. VitD deficiency is common in aged individuals, and has been proposed as an independent indicator of frailty and mortality [13]. Recently, VitD deficiency has been shown to result in muscle wasting with increased protein breakdown due to hyperactivation of the ubiquitin-proteasome pathway [14]. Consistent with a protective role of VitD in muscle, VitD supplementation in elderly patients has been shown to improve skeletal muscle functions, such as increased lower limb muscle strength [15] and reduction in the relative risk of falls [16]. Nevertheless, a number of studies have failed to demonstrate an effect of VitD on physical performance [17, 18], a matter that is still controversial [19].

VitD effects on muscle cells are long known [20], and several observational studies have been performed on the role of VitD on skeletal muscle. VDR knock-out mice have provided evidences for a direct role of this steroid receptor in the skeletal muscle. Endo and coworkers (2003) have demonstrated that myofibers in VDR-null mutant mice are smaller than in wild type mice. Moreover, they showed that skeletal muscles from 3 weeks old mice lacking VDR display an abnormally elevated expression of myogenic factors such as Myogenin, Myf5 and E2A, as well as increased expression of embryonic and neonatal myosin heavy chain (MyHC; [21]). Taken together, these data suggest that VDR is an important regulator of skeletal muscle development and differentiation.

Despite evidence demonstrating that VitD acts on the skeletal muscle, the effectiveness of VitD administration in pathological conditions characterized by muscle atrophy has not been assessed. For this reason, the present study was designed to investigate: i) the role of VitD in the pathogenesis of cancer cachexia; ii) the possibility to use VitD treatment to prevent muscle wasting in an experimental model of cancer cachexia (rats bearing the AH130 Yoshida ascites hepatoma); iii) the mechanisms by which vitamin D might affect muscle phenotype in tumor-bearing animals. The results obtained show that, while VitD does not significantly improve cachexia, it does affect myogenesis.

## RESULTS

### VitD administration did not prevent cachexia in the AH130 hosts

**Figure 1:**
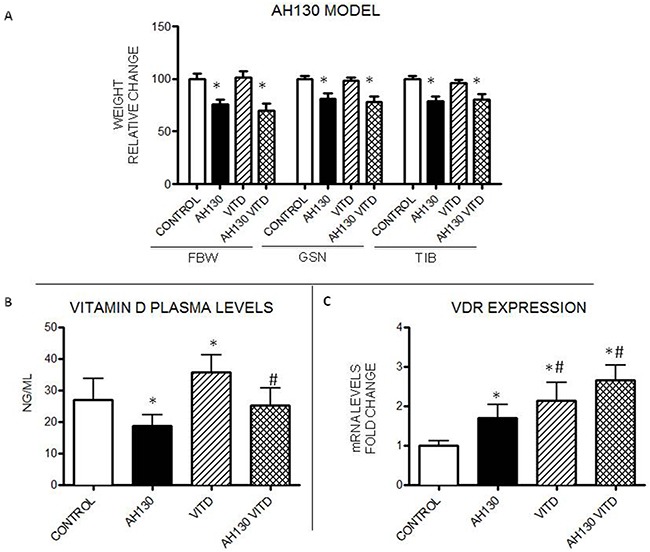
Effects of VitD3 administration to rats bearing the AH130 hepatoma **A**. Body weight changes, gastrocnemius and tibialis muscle weight in controls and tumor-bearing rats either untreated or receiving VitD3. **B**. VitD plasma levels in untreated and VitD3-treated rats. **C**. VDR mRNA expression levels in the tibialis muscle of untreated and VitD3-treated animals. Data (means±SEM) are expressed as fold change (control rats: n=6, tumor-bearing rats: n=8). Significance of the differences: *p< 0.05 *vs* control; #p< 0.05 *vs* AH130.

Rats bearing the AH130 hepatoma represent a well characterized model for the study of cancer cachexia, showing a progressive loss of body weight that parallels tumor growth [22]. Body weight loss in the AH130 bearers is associated with severe depletion of skeletal muscle mass (Figure 1A) and with significant reduction of VitD plasma levels below control values (Figure 1B). Low circulating VitD, is paralleled by a significant increase of VDR mRNA in the skeletal muscle of the AH130 hosts (Figure 1C).

The administration of VitD3 (80 IU/kg, daily intragastrical administration, starting the day of transplantation) increases 25(OH)-VitD circulating levels (about 35% above baseline) in both controls and AH130 hosts (Figure 1B). However, no appreciable effects on tumor mass (data not shown), final body weight (FBW), gastrocnemius and tibialis muscle mass (GSN and TIB respectively) can be observed in treated *vs* untreated animals (Figure 1A). Consistent with previous *in vitro* and *in vivo* observations [23], VitD3 supplementation induced an increase above baseline of VDR mRNA expression in the tibialis muscle of control animals (confirming data by Girgis et al [24]) and further exacerbated the increase occurring in the AH130 hosts (Figure 1C).

### Muscle VDR overexpression was observed in tumor hosts

To ascertain that VDR overexpression in the skeletal muscle is not a peculiar feature of the AH130-bearing rats, VDR levels were assessed also in mice transplanted with the Lewis lung carcinoma (LLC) or with the Colon 26 carcinoma (C26), both being able to induce cachexia in the host mouse [6, 25].

**Figure 2:**
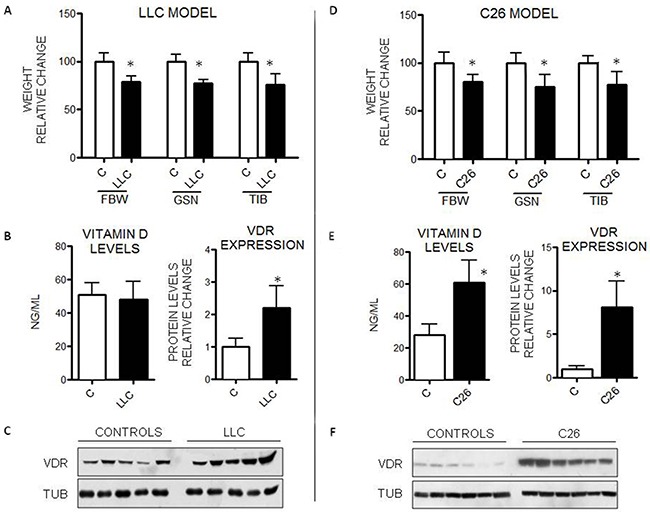
VitD levels and VDR expression in mice bearing the LLC or the C26 tumor **A, D**. Final body weight (FBW), gastrocnemius (GSN) and tibialis (TIB) mass in controls (n=5) and tumor-bearing mice (n=7). **B, E**. Circulating VitD and VDR protein expression levels in GSN muscle. **C, F**. Representative WB analysis of VDR expression in the gastrocnemius (anti-VDR antibody, clone D6, Santa Cruz Biotechnology). Data are means±SD. Significance of the differences: *p< 0.05 *vs* C.

Mice injected with LLC cells show a significant decrease in body weight and skeletal muscle mass, as reported in Figure 2A. Although 25(OH)-VitD plasma levels were not significantly different between controls and tumor-bearing mice (Figure 2B), VDR protein expression was increased in the skeletal muscle of the latter (Figure 2B, 2C).

C26 tumor growth is associated with a marked loss of body weight, as well as of both gastrocnemius and tibialis muscle mass (Figure 2D). Both 25(OH)-VitD levels and VDR expression were increased in the C26 bearers with respect to control mice (Figures 2E, 2F). 25(OH)-VitD circulating levels were markedly lower in control Balb/c mice than in control C57BL/6J animals, conforming to previous data [26]. The administration of VitD3 to the C26 hosts produced results comparable to those reported in rats bearing the AH-130 hepatoma (Supplementary Figure 1).

Finally, VDR mRNA expression was also increased in skeletal muscle biopsies of cancer patients with respect to those of control subjects (Supplementary Figure 2).

### Muscle VDR levels regulate myogenic differentiation

The VDR signaling pathway has been linked to defects in skeletal muscle development [21]. To investigate this point, the effects of VitD treatment were studied *in vitro* on C2C12 myocyte cultures. VitD addition to the culture medium slowed C2C12 myoblast proliferation in a dose- and VDR-dependent manner (data not shown), confirming previous observations [24, 27]. Moreover, attempts to produce VDR overexpressing C2C12 myoblasts by gene transfection were revealed to be unsuccessful, suggesting that high VDR levels might be toxic, in myoblasts at least, lowering proliferation rates and eventually leading to cell death (data not shown).

**Figure 3:**
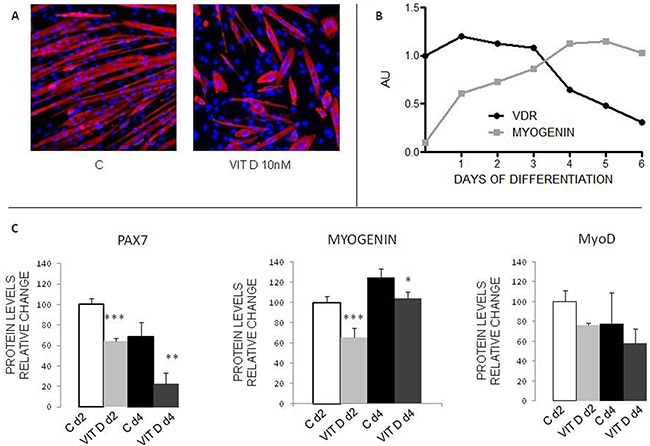
Effects of VitD on C2C12 myoblast differentiation **A**. Immunostaining of control or VitD-treated C2C12 cells after 4 days of differentiation (Red: MyHC, Blue: nuclei). **B**. Expression levels of VDR (black line) and myogenin (grey line) during C2C12 myoblast differentiation in the absence of VitD treatment, as measured by western blotting analysis (Santa Cruz Biotechnology: anti-VDR antibody, clone D6; anti-myogenin antibody, clone F5D). **C**. Protein expression levels of some myogenic regulatory factors in control or VitD-stimulated C2C12 cells at day 2 and 4 of differentiation (Santa Cruz Biotechnology: anti-myogenin antibody, clone F5D, anti-MyoD antibody, clone M318; Developmental Studies Hybridoma Bank, University of Iowa: anti-Pax7 antibody). Data (means±SD) are expressed as % of controls. Significance of the differences: ***p<0.001, **p<0.01, *p<0.05 *vs* C, 3 independent experiments.

**Figure 4:**
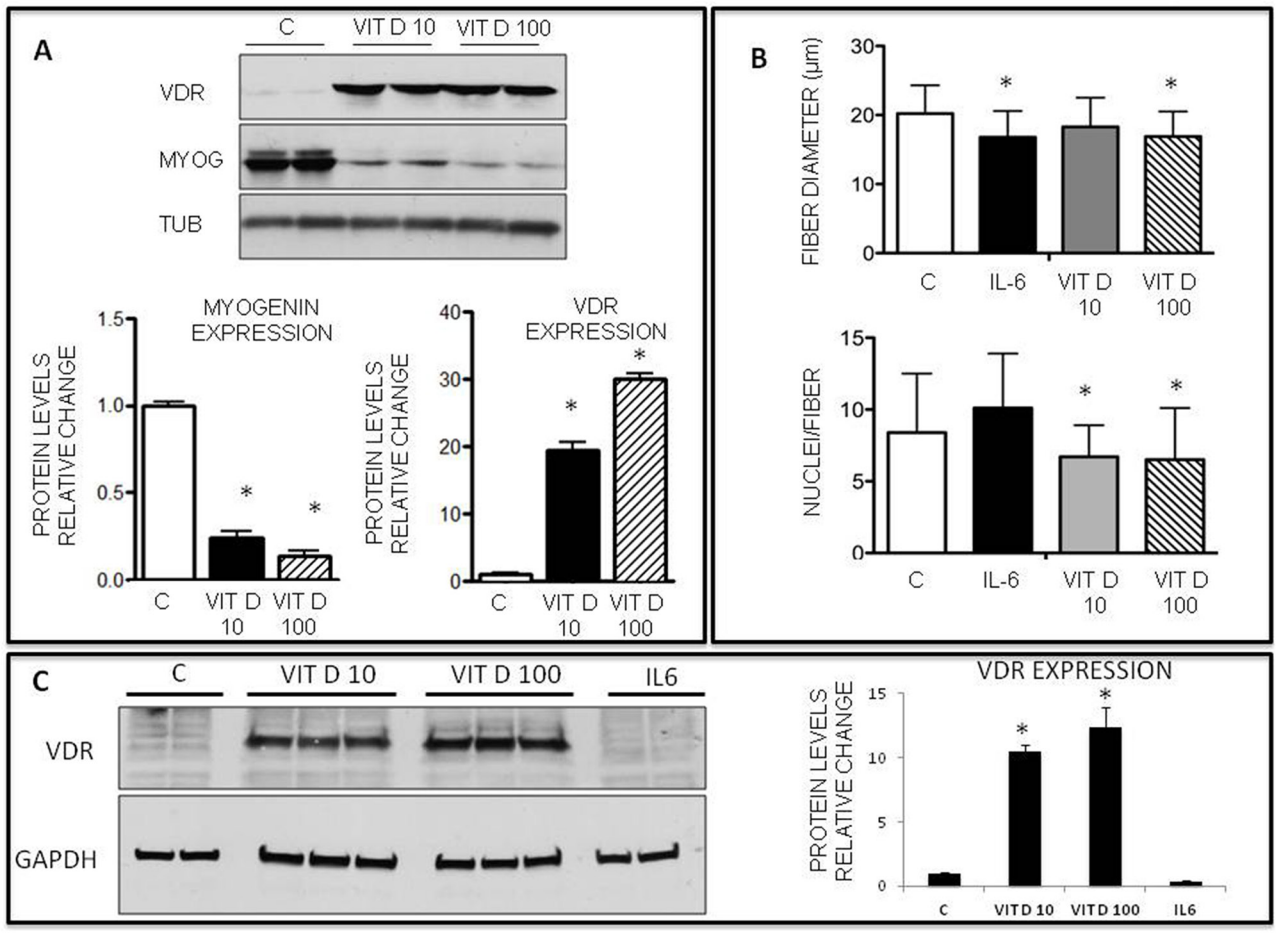
Effects of VitD on VDR and myogenin expression on C2C12 differentiating myoblasts and on myotubes **A**. Protein expression levels of VDR and myogenin in control or VitD-stimulated C2C12 cells at day 4 of differentiation (Santa Cruz Biotechnology: anti-VDR antibody, clone D6, anti-myogenin antibody, clone F5D); **B**. Average myotube diameter and average number of nuclei/myotube in C2C12 cultures treated with control medium or with medium containing: 100ng/ml IL6, 10nM or 100nM VitD (bars show the average of three independent experiments, n=100 for each condition); **C**. Western blotting analysis of representative samples showing VDR expression (Santa Cruz Biotechnology: anti-VDR antibody, clone D6). Data are means ± SD. Significance of the differences: *p< 0.05 vs C, 3 independent experiments.

To test if VitD could affect myogenic differentiation, C2C12 myoblasts were grown to confluence and then induced to differentiate in the appropriate medium, with or without 10 nM 1.25-OH^2^-vitD for 4 days. As demonstrated by immunofluorescence for MyHC (Figure 3A), control myoblasts were able to fuse together and to form myotubes with the canonical elongated structure, while VitD treated cells only partially fused (avarage number of nuclei/myotube: C=6.26±1.99, VitD=3.33±1.56, p<0.05, n=200. Total number of nuclei: C=83.66±13.01, VitD=58.66±14.67, p=0.09, n=3) and give rise to multinucleated structures with aberrant shape and low MyHC content (Figure 3A and Supplementary Figure 3A). Western blotting analysis showed a rapid decline of VDR levels as differentiation progressed; such reduction is paralleled by increased levels of myogenin, one of the markers of myogenic differentiation (Figure 3B, Supplementary Figure 3B). When differentiation was induced in the presence of VitD, the levels of myogenic regulatory factors such as Pax7 and myogenin were significantly reduced, at both 2 and 4 days after shifting to differentiation medium, while the expression of MyoD was not different from controls (Figure 3C, Supplementary Figure 3C). At the same time, in parallel to myogenin reduction, VDR was overexpressed at both mRNA (Supplementary Figure 3D) and protein level (Figure 4A), in a negatively correlated manner (r^2^=0.89, p-value = 0.0031).

To ascertain if VitD treatment could also affect mature myotubes, C2C12 myoblasts were differentiated for 4 days in regular differentiation medium and then treated with for 48 h with VitD (10-100 nM) or with IL-6, as a positive control of fiber shrinkage [28]. The results show that only 100 nM VitD significantly decreased myotube size (Figure 4B), while the number of nuclei per myotube was significantly reduced by both VitD concentrations (Figure 4B). Interestingly, although IL-6 treatment was comparable to 100 nM VitD as for myotube shrinkage, it did not affect the number of nuclei per myotube (Figure 4B), suggesting that two different mechanisms are responsible for VitD and IL-6-induced decrease of myotube size. Finally, VitD, but not IL-6, treatment of C2C12 myotubes markedly increased VDR expression (Figure 4C).

**Figure 5:**
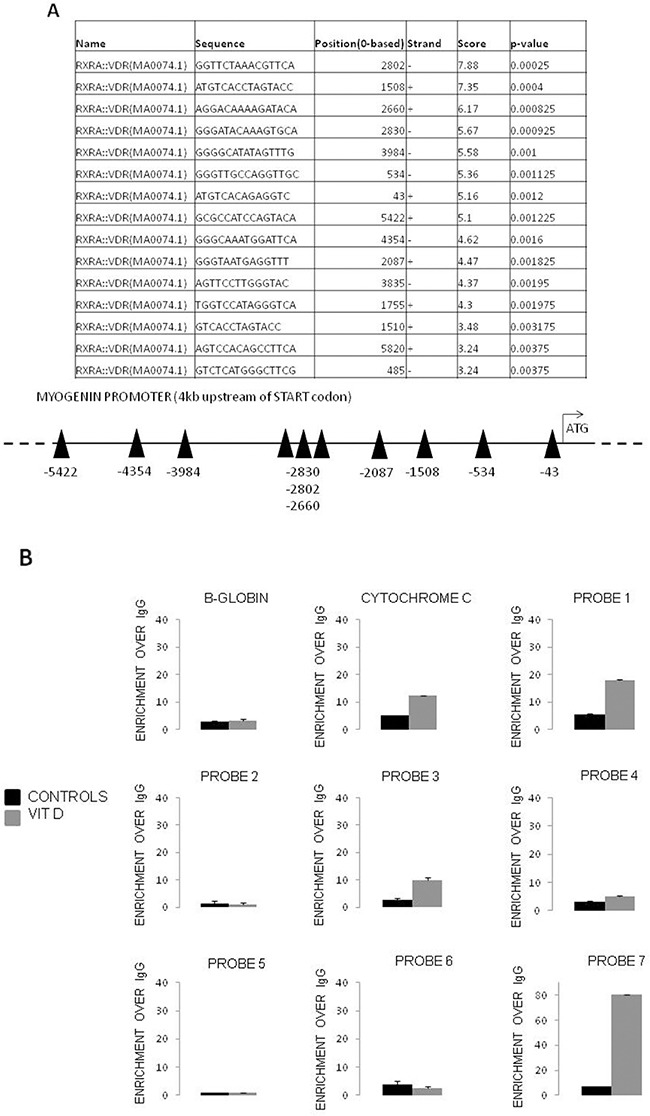
VDR is recruited at regulatory regions of the myogenin gene after VitD exposition **A**. Putative VDR binding sites in the promoter region of the Myogenin gene (6.5 Kbp), as predicted by LASAGNA-Search web tool, and schematic representation of the Myogenin promoter region showing putative binding sites for VDR; **B**. ChIP assay (representative pattern) was performed with chromatin extracted from C2C12 myoblasts treated or untreated with VitD, using normal rabbit IgG and an anti-VDR antibody. β-globin and cytochrome c promoter regions were amplified as negative and positive controls, respectively.

The observation reported above that myogenin and VDR expression are negatively correlated suggested the occurrence of a direct transcriptional regulator activity of VDR. To investigate this point, the promoter region of the mouse myogenin gene was analyzed *in silico* for the presence of putative VDRE. Figure 5A shows the presence of 15 predicted VDR responsive elements in the 10kb upstream myogenin transcription start, six of which placed in the last 5kb upstream the coding region. Chromatin immunoprecipitation was performed to validate the *in silico* analysis. The results demonstrate that three among the putative binding sites exhibited an enrichment after immunoprecipitation with anti-VDR antibody, demonstrating a direct interaction of VDR with the myogenin promoter (Probes 1, 3, 7; Figure 5B).

### VDR silencing restores C2C12 myoblast differentiation

**Figure 6:**
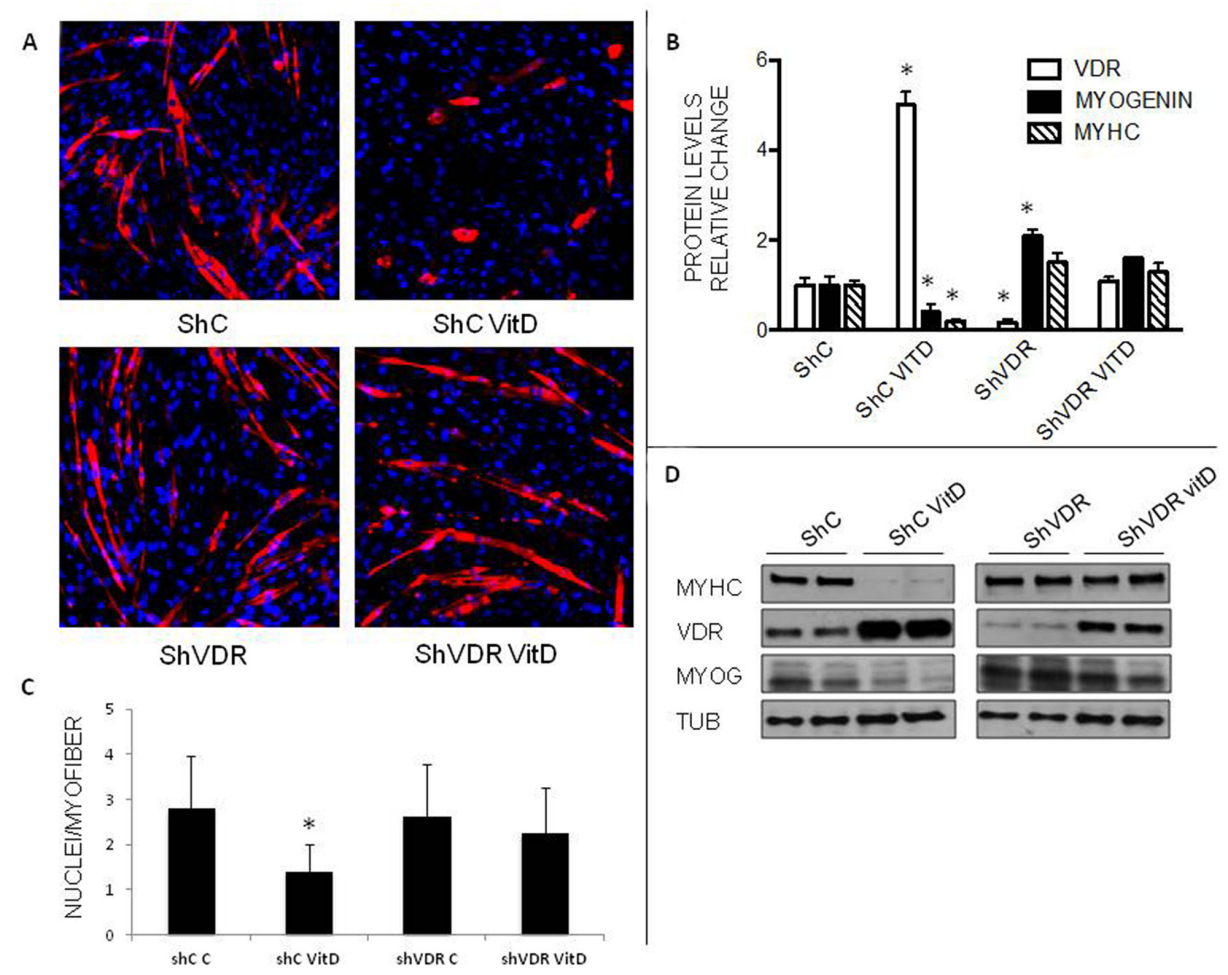
Effects of VitD treatment on VDR-silenced C2C12 cells **A**. Immunostaining of C2C12 cells after 4 days of differentiation (Red: MyHC, Blue: nuclei). **B**. Protein expression levels of VDR, MyHC and myogenin at day 4 of differentiation. Data (means±SD) are expressed as relative change. **C**. number of nuclei/myofiber. **D**. representative western blotting pattern of MyHC, myogenin and VDR expression (Santa Cruz Biotechnology: anti-VDR antibody, clone D6, anti-myogenin antibody, clone F5D; Sigma: anti MyHC antibody, clone MY32). Significance of the differences: *p<0.05 *vs* ShC, 2 independent experiments.

To test the relevance of VDR to myogenesis, its expression was silenced by means of lentivirus-delivered specific shRNA. In this condition (shVDR), C2C12 cells were no longer sensitive to VitD treatment and differentiated normally into mature myotubes (Figure 6A), as also indicated by the number of nuclei/myotube (Figure 6C). Figure 6B shows that VDR silencing was robust (-83% in shVDR cells compared to shC) and that the marked reduction of both myogenin and MyHC expression induced by VitD treatment in ShC infected cells was not detectable in shVDR cultures (Figure 6C). Intriguingly, untreated ShVDR cultures expressed more myogenin and MyHC than untreated ShC cells (Figure 6B, 6C), suggesting that the lack of VDR enhanced myogenic differentiation even in basal conditions. When shVDR cells were stimulated with 10 nM VitD, the expression levels of both VDR and myogenin were at least as high as in ShC untreated cells.

### VitD administration impairs muscle regeneration *in vivo*

**Figure 7:**
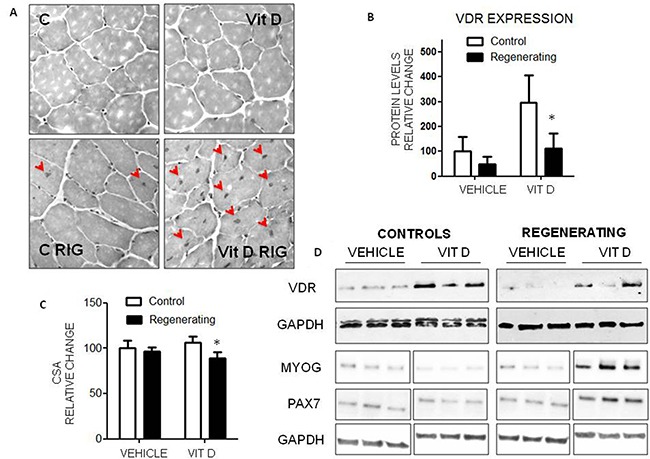
Effects of VitD administration during muscle regeneration **A**. Histological pattern of regenerating and non regenerating muscles in VitD3-treated (n = 6) and untreated mice (n = 6). For experimental details see Materials and Methods. **B**. VDR expression in regenerating and non regenerating muscles in the presence or in the absence of VitD3 treatment (Santa Cruz Biotechnology: anti-VDR antibody, clone D6). Data (means±SD) are expressed as relative change. Significance of the differences: *p< 0.05 *vs* C. **C**. Average myofiber CSA of controls (white bars) or regenerating (black bars) muscles 15 days after BaCl_2_ injection; 400 myofibers (regenerating or not)/condition have been counted. **D**. Representative samples showing VDR, Pax7 and myogenin expression (Santa Cruz Biotechnology: anti-VDR antibody, clone D6, anti-myogenin antibody, clone F5D; Developmental Studies Hybridoma Bank, University of Iowa: anti-Pax7 antibody).

To test whether the anti-differentiation effects mediated by VitD and VDR *in vitro* occur also *in vivo*, VitD3 (80 IU/kg/day) was administered to animals in which muscle injury has been induced by BaCl_2_ injection in the *tibialis anterior* muscle. As described in the literature, BaCl_2_ causes an extensive muscle injury and the subsequent regeneration involves about 90% of muscle fibers [29]. In untreated mice, total fiber CSA as well as the number of fiber with central nuclei, evaluated 15 days after damage, were not significantly different between injured and uninjured muscles, suggesting that complete regeneration had already occurred (Figures 7A, 7C). By contrast, and consistent with the *in vitro* results, in the damaged muscle, the CSA of VitD3-treated animals was decreased, while the number of fibers with central nuclei was higher than in the controlateral uninjured one (Figures 7A, 7C). In VitD3-supplemented mice VDR expression was markedly increased in the uninjured muscle, while it remained comparable to vehicle-treated animals in the regenerating one (Figure 7B and 7D). The expression level of myogenin and Pax7 were consistent with the impaired/delayed regeneration (Figure 7D). Finally, VitD3 administration proved unable to improve muscle wasting also in experimental arthritis (Supplementary Figure 4), a condition previously shown to be associated with enhanced muscle regeneration [30].

## DISCUSSION

The present study is the first to investigate the involvement of VitD in the pathogenesis of cancer cachexia. Low VitD levels are a frequent feature in advanced cancer patients with cachexia and fatigue [31], while high serum VitD has been associated with low mortality in colorectal cancer patients [32]. However, data reported in the present study demonstrate that, depending on the experimental model of cancer cachexia chosen, circulating VitD levels may be reduced (AH130 hosts), unchanged (LLC hosts) or increased (C26 hosts) compared to control values. Irrespective of circulating VitD, however, the presence of the tumor was always associated with up-regulation of muscle VDR expression. This *in vivo* observation, coupled with the results obtained *in vitro*, suggests that VDR might play a role in the onset and/or progression of cachexia.

Quite recent observations show that VitD supplementation in patients with non-metastatic HER2^+^ breast cancer treated with trastuzumab is associated with improved disease-free survival and with a tendency to increase body mass index [33]. Along this line, to assess if VitD supplementation could affect cancer cachexia, rats bearing the AH130 tumor, characterized by low VitD plasma levels, were treated with this hormone. The results show that body weight and muscle mass did not appreciably change in VitD-treated *vs* untreated tumor hosts, although VitD plasma levels in the former were nearly restored to control values. Of interest, in the AH130 hosts treated with VitD, muscle VDR levels were higher than those detected in the untreated tumor-bearing rats, likely in view of the restoration of normal circulating vitamin levels. In this regard, it is well known that VitD positively regulates the expression of its main receptor both *in vivo* and *in vitro* [34, 35]. The other way round, the present study shows that increased VDR levels in the muscle of tumor-bearing animals seems a constant finding, irrespective of changes in circulating VitD levels. On the whole, these observations suggest that: i) a direct link between VitD plasma levels and the outcome of cancer cachexia is lacking; and ii) a ligand-independent dysregulation of VDR signaling pathway could be involved in the pathogenesis of cancer-induced muscle wasting. In this regard, in certain conditions VDR has been proposed to act independently of VitD, although it still requires functional VitD responsive elements as well as an intact VDR DNA binding domain; the effects deriving from such action are thus indistinguishable from VitD-dependent transactivation [36]. On this line, VDR up-regulation in the skeletal muscle of tumor hosts could be considered *per se* as a condition sufficient for its transcriptional activity. Consistent with such a mechanism, a VitD-independent, toll-like receptor (TLR)2-dependent VDR hyperactivation has been proposed to contribute to the regulation of innate immune response [37].

The observation that VDR is overexpressed in the skeletal muscle of tumor-bearing animals suggests that the VitD/VDR-dependent signaling pathway might contribute to cancer-induced muscle wasting, despite the lack of effect of VitD supplementation. In this regard, VDR relevance to skeletal muscle biology has been previously described. As an example, Endo and coworkers reported that VDR knock-out mice develop muscles smaller than those of wild-type animals, with high expression levels of myogenic regulatory factors (MRF) such as Myogenin, Myf5 and E2A. Moreover, the authors showed that MRF expression in myoblast cultures is decreased by VitD treatment. An opposite view is reported by a study showing that MRF expression is reduced in VitD deficient rats [14].

The results obtained in the present study on C2C12 and primary myoblasts clearly show that VitD treatment impairs their differentiation into mature myotubes, likely due to the modulation of both VDR (increased) and myogenin (decreased) expression. In this regard, these observations are consistent with recent data by Girgis and coworkers showing that VitD inhibits myotube formation in C2C12 cultures and that the few formed myotubes appeared bigger than those obtained in control C2C12 myocytes. The authors ascribed this latter effect to modulations of the myostatin-dependent pathway. No changes in myotube size were detected in the present study, however; the different experimental period (10 days in Girgis et al., 6 days in the present study) could account for such a discrepancy. The inhibition of myotube formation induced by vitD suggests an impairment of myogenesis. Consistent with this hypothesis, muscle regeneration after BaCl_2_-induced damage was altered in VitD-treated mice. The relevance of VDR to myogenic impairment in VitD-treated cells is demonstrated by the observation that normal differentiation was restored when VDR expression was silenced. Of interest, also in the absence of VitD stimulation, myogenin levels were higher in VDR-silenced than in wild-type C2C12 cells, strengthening the hypothesis of a ligand-independent, VDR-mediated, negative regulation of myogenin transcription. Finally, the presence of several VDRE in the promoter region of the myogenin gene and the demonstration that VDR can directly bind the myogenin promoter support the proposed mechanism of regulation.

VDR expression in C2C12 cells is high at the beginning of the differentiation process and is progressively reduced until the cells complete their maturation into myotubes. This observation is consistent with previous data reported in the literature, showing that mean intracellular VDR content is higher in undifferentiated than in differentiated cells [35, 38]. Moreover, VDR expression is sustained in myoblasts of different origins as well as in the developing skeletal muscle [21, 39, 40]. By contrast, VDR expression in the adult muscle or in differentiated myotubes is always reported as very low or even absent [41], although the results shown in the present study demonstrate that it can be stimulated by VitD treatment, while not by the proinflammatory cytokine IL-6. While the atrophy-inducing properties of IL-6 are well described, little is known about the possibility that VitD, likely through VDR, can stimulate myonuclei loss and/or MyHC degradation in differentiated myotubes. In this regard, however, the data shown in the present study are more likely representative of an inhibition of still ongoing myonuclei accretion. Indeed, C2C12 cultures are not completely differentiated after 4 days in differentiation medium (see Figure 3A). In addition, the suggestion that VitD does not stimulate protein degradation is supported by a previous report showing that protein synthesis induced by leucine and insulin in C2C12 myotubes is enhanced in the presence of 10 nM calcitriol [42].

Taken together, these considerations highlight that VDR-dependent signaling pathway is likely to participate in the regulation of the first phases of myogenic differentiation *in vitro* and of skeletal muscle development and regeneration *in vivo*. In this regard, VDR down-regulation may represent a condition required to achieve complete myogenic differentiation. Such a hypothesis is supported by the results in this study showing that animals administered VitD display an impaired muscle regeneration that is associated with a tendency to increase VDR expression. These results are in agreement with a very recent study showing that a supraphysiological dose of VitD injected into damaged muscle (days 4-7 after BaCl_2_) delays the regenerative response [43]. The experimental setting shown in the present study, however, is quite different: the animals received VitD3, the administration route was intragastric, and treatment started the day of injury to finish the day before sacrifice.

An altered myogenic potential has been shown to contribute to cancer-induced muscle wasting [5, 6]. Activation of the MAPK/ERK pathway plays a role in this regard, contributing to keep Pax7^+^ cells from proceeding along differentiation, leading to their accumulation into the skeletal muscle [6]. The results shown in the present study suggest that also VDR overexpression could contribute to the impaired myogenesis observed in tumor hosts, mainly by altering the expression of myogenic factors involved in the differentiation program, such as myogenin [6]. Interestingly, an interaction between ERK and VDR signaling pathways has previously been proposed: in MG-63 (human osteoblastic osteosarcoma cell line) and HeLa (human cervical carcinoma cell line) cells, ERK activation leads to increased VDR transcriptional activity through overexpression of its coreceptor RXR gamma [44]. In this regard, ERK activation occurring in the skeletal muscle of cachectic tumor-bearing animals could be responsible for the establishment of a ‘VDR-prone’ environment, resulting in enhanced VDR-dependent signaling.

It is also worth citing that a relationship between VDR activation and skeletal muscle disorders has already been reported. The FokI polymorphism of VDR gene is a T/C transition in the second exon, resulting in a truncated protein (424aa instead of 427aa) with enhanced transactivation capacity [45]. Two studies in humans suggest that FokI polymorphism is associated with decreased skeletal muscle mass and strength. In particular, Roth and coworkers showed that FokI homozygous men display a low fat-free muscle mass and a risk of sarcopenia 2.17-fold higher than controls [46]. The other study demonstrates that homozygosity for the FokI polymorphism is associated with reduced quadriceps strength compared with heterozygosity or control subjects [47]. Finally, a recent study on cancer patients shows a correlation between the presence of specific VDR BsmI and TaqI alleles and CRP plasma levels, one marker of cancer cachexia [48]. In particular, the fact that VDR b and T alleles are more frequent in cachectic cancer patients with elevated CRP levels leads to the conclusion that this may represent an early clinical predictor for more aggressive forms of cachexia [49]. Intriguingly, the VDR bT genotype has been previously demonstrated to be associated with high expression levels of both VDR mRNA and protein [45, 50–52].

In conclusion, the present study suggests that VDR overexpression in animal models of cancer cachexia is likely contributing to skeletal muscle wasting through an impairment of the muscle regenerative program. An important limitation of the present study is the lack of a vitamin D-depleted control. Indeed, most of the clinical trials reported in the literature describe the effects of vitamin D supplementation in a context of vitamin D deficiency, while at least in part the present data reflect the effects of supraphysiological vitamin D levels. Another point is that vitamin D-induced VDR overexpression in the AH130 hosts does not worsen muscle wasting, in some way opposing to the potential involvement of VDR in causing cancer-induced muscle depletion. However, muscle weight loss in the AH-130 hosts is rather severe, and the possibility that it cannot be further exacerbated should be taken into account.

On the basis of the results shown in the present study we can then postulate that a note of caution should be made when considering VitD supplementation in patients affected by cancer cachexia or any other condition of skeletal muscle atrophy where a regenerative process might be involved. In this regard, too much circulating VitD may cause a further increase in the expression levels of VDR that could decrease the regenerative potential of skeletal muscle precursor cells. Along this line, the decrease in VitD circulating levels observed in the AH130 hosts as well as in cancer patients [31] could represent a compensatory mechanism aimed at counteracting the increased VDR expression in the skeletal muscle. However, another possibility is that VitD plasma levels could be modulated independently from muscle VDR, for example by modulations of VitD-binding proteins. Finally, our *in vitro* experiments suggest that VDR is likely to play a fundamental role during myoblast differentiation, regulating and timing the expression of important myogenic factors, such as myogenin.

## MATERIALS AND METHODS

### Reagents

All reagents supplied by Sigma-Aldrich (St. Louis, MO, USA), unless differently specified.

### *In vivo* experiments

Experimental animals were cared for in compliance with the Italian Ministry of Health Guidelines and the Policy on Humane Care and Use of Laboratory Animals (NIH, 1996). The experimental protocol was approved by the Bioethical Committee of the University of Torino (Torino, Italy). Male Wistar rats weighing approximately 150 g, Balb-c and C57/BL6 mice weighing approximately 20 g were obtained from Charles River (Calco, Italy) and were maintained on a regular dark-light cycle (light from 8 a.m. to 8 p.m.), with free access to food and water during the whole experimental period. Rats were injected intraperitoneally with 10^8^ AH130 Yoshida ascites hepatoma cells [a gift many years ago from Prof. Ugo Del Monte (University of Milano, Milano, Italy) and maintained in our laboratory by weekly i.p. transplantation], whereas tumor-bearing mice (n= 8) were subcutaneously inoculated between the shoulder blades with 5 × 10^5^ Colon 26 carcinoma cells [a gift from Prof. Mario P. Colombo (Istituto di Ricovero e Cura a Carattere Scientifico National Cancer Institute, Milano, Italy)] or with 5 × 10^5^ Lewis Lung Carcinoma cells (ATCC, Manassas, VA). C26 and LLC cells were expanded to 50,000/cm^2^ *in vitro* in Dulbecco's modified Eagle's medium (DMEM) supplemented with 10% fetal bovine serum, 100 U/mL penicillin, 100 μg/mL streptomycin, 100 μg/mL sodium pyruvate, and 2 mmol/L L-glutamine. Cells were maintained at 37°C in a humidified atmosphere of 5% CO_2_ in air, detached with trypsin, resuspended in sterile saline, and subsequently implanted in the animals at the concentrations indicated above. Rats or mice inoculated with vehicle (saline) served as controls (n= 6). In the AH130 experiment, animals were randomized and divided into four groups: controls (n= 6) and AH130 (n= 8), administered or not VitD3. Treated groups received intragastrically a daily administration of 80 IU/kg body weight (b.w.) of VitD3 dissolved in 200 μl of corn oil. Control rats were administered with an even amount of corn oil. The same treatment schedule was also applied to a group of mice, divided into controls (C) and C26 hosts (C26), treated or untreated with VitD3. VitD3 dosage was selected on the basis of data reported in the literature as well as by performing dose-response preliminary experiments in control animals, having VDR as endpoint. The intragastrical route of administration was chosen taking into consideration a potential translation to the clinical practice.

Animal weight and food intake were recorded daily. AH130-bearing rats, C26 hosts and LLC-bearing mice were euthanized under anesthesia 7, 14 and 28 days after tumor transplantation, respectively. Several muscles and organs were rapidly excised, weighed, frozen in isopentane cooled with liquid N_2_, and stored at −80 °C.

In the regeneration experiment, skeletal muscle injury was induced in 8 week old mice by injecting 1.2% BaCl_2_ (30 μl) into the left *tibialis anterior* muscle. The controlateral muscle was injected with PBS and used as control. Half of mice (n=6) received VitD as described above. After 15 days of recovery the animals were euthanized and the tibialis muscles were dissected and removed, rapidly frozen in melting isopentane and used for cross-sectional area (CSA) and protein expression analysis.

Healthy or arthritic male Wistar rats (150 g/6 weeks old) were purchased from Charles River. An intradermal injection of heat-inactivated *Mycobacterium butyricum* (4 mg) induced arthritis in the right paw of rats. Controls received just vehicle (paraffin oil, 100 μl). Animals were maintained on a regular dark-light cycle (light from 8 a.m. to 8 p.m.), with free access to food and water during the whole experimental period. Arthritic and control rats were divided into two groups: VitD treated (80 IU/kg b.w./day, intragastrical administration) or vehicle (corn oil) treated (sham). Body weight and arthritis severity were assessed daily. Evaluation of the latter was performed by measuring the arthritis index of each animal, which was scored by grading each paw from 0 to 4, since inflammation of the paw is associated with radiological and histological alterations of the joints ([53]). After 15 days, rats were sacrificed and muscles (gastrocnemius, tibialis anterior and heart), liver and spleen were rapidly excised, weighed, frozen in liquid nitrogen, and stored at –80°C for further analysis.

### Human samples

Already available samples, deriving from a previous study [54], were used. Muscle biopsies were obtained from patients recruited at the M.G. Vannini Hospital in Rome (Italy) after signing an informed consent and after clearance by the local Ethical Committee. Briefly, patients undergoing abdominal surgery for cancer (mainly gastrointestinal tumors) or for non neoplastic reasons (used as controls) were included. The following exclusion criteria were adopted: liver failure, diabetes, metabolic acidosis, acute and chronic renal failure, sepsis, AIDS, inflammatory bowel diseases, acute and chronic hepatitis, autoimmune disorders and chronic obstructive pulmonary disease. Biopsy specimens were obtained during the initial phase of the operation from the *rectus abdominis* muscle (for details see [54]), immediately frozen in liquid nitrogen and stored at - 80 °C until analysis.

### Vitamin D concentration

For the determination of VitD circulating levels, blood samples were centrifuged at 600*g* for 10 min and the separated plasma was stored at −20°C. 25D plasma levels were determined by radioimmunoassay (Immunodiagnostic Systems Holdings, Boldon, UK).

### Histological analysis

Serial 10 μm-thick frozen sections were cut from cryopreserved tissue blocks, adhered to Superfrost Plus microscopy slides and stained with hematoxylin and eosin. All sections were examined by light microscopy (Nikon Eclipse TS100) and digital images were obtained with a Nikon COOLPIX 4500 camera. To assess myofiber CSA 400 fibers of gastrocnemius muscle sections were counted and measured using Image J software (http://rsb.info.nih.gov/ij/; NIH, Bethesda, MD).

### Cell cultures

Murine C2C12 skeletal myoblasts (ATCC) were grown in high-glucose DMEM supplemented with 10% fetal bovine serum, 100 U/mL penicillin, 100 μg/mL streptomycin, 100 μg/mL sodium pyruvate, and 2 mmol/L l-glutamine and maintained at 37°C in a humidified atmosphere of 5% CO_2_ in air. Differentiation was induced by switching subconfluent cultures to DMEM supplemented with 2% horse serum (differentiation medium). In the differentiation experiments VitD (calcitriol; 1,25(OH)2 D dissolved in DMSO) was added to the medium at a final concentration of 10 nM or 100 nM, starting from the first day of differentiation. Since VitD is known to have a short half life at 37°C (5/8 hours), the medium was replaced every day during the experiments. Control cells received an equal amount of DMSO.

### Gene silencing

VDR silencing was achieved by using shRNA. A set of four MISSION shRNAs targeting VDR mRNA and cloned into plKO.1-puro expression vector was used. Third generation lentiviral particles (kindly provided by Prof. Ponzetto, CERMS - University of Torino, Torino, Italy) were used to deliver the shRNA to C2C12 cells. Cells were then incubated in their regular growth medium containing Puromycin 1μg/ml, in order to select for stably transformed cells. After testing the expression of VDR via western blotting analysis, cells infected with the shRNA providing the highest silencing were used for all the experiments (5′-CGGCCTGAGATCAATCACATTTAACTCGAGTTAAATGTGATTGATCTCAGGTTTTT-3′). Control cells were infected with a plKO.1-puro plasmid containing a non-specific shRNA (MISSION pLKO.1-puro Non-Mammalian shRNA Control Plasmid DNA).

### Western blotting analysis

Approximately 50 mg of gastrocnemius or tibialis muscle were homogenized in 80 mmol/L Tris-HCl, pH 6.8, containing 100 mmol/L dithiothreitol, 70 mmol/L SDS, and 1 mmol/L glycerol, with freshly added protease and phosphatase inhibitor cocktails; kept on ice for 30 minutes; centrifuged at 15,000 ×*g* for 10 minutes at 4°C and the supernatant collected. Protein concentration was assayed according to Bradford, using bovine serum albumin as working standard. C2C12 cells were lysed on RIPA buffer (50 mmol/L Tris-HCl, pH 7.4, 150 mmol/L NaCl, 1% Nonidet P-40, 0.25% sodium deoxycholate, 1 mmol/L phenylmethylsulfonyl fluoride) with freshly added protease and phosphatase inhibitor cocktails. Equal amounts of protein (30 μg) were heat-denaturated in sample-loading buffer (50 mmol/L Tris-HCl, pH 6.8, 100 mmol/L dithiothreitol, 2% SDS, 0.1% bromophenol blue, 10% glycerol), resolved by SDS-PAGE, and transferred to nitrocellulose membranes (Bio-Rad Laboratories, Hercules, CA). The filters were blocked with Tris-buffered saline containing 0.05% Tween and 5% nonfat dry milk and then were incubated overnight with antibodies directed against VDR (mouse monoclonal antibody, clone D6, Santa Cruz Biotechnology, Santa Cruz, CA), Myogenin (mouse monoclonal antibody, clone F5D, Santa Cruz Biotechnology, Santa Cruz, CA), MyHC (mouse monoclonal antibody, clone MY32), Pax7 (Developmental Studies Hybridoma Bank, University of Iowa), MyoD (rabbit polyclonal antibody, clone M318, Santa Cruz Biotechnology, Santa Cruz, CA), GAPDH (goat polyclonal antibody, V18, Santa Cruz Biotechnology, Santa Cruz, CA) and tubulin (mouse monoclonal antibody, clone T5168). Peroxidase-conjugated IgGs (Bio-Rad Laboratories) were used as secondary antibodies. Quantification of the bands was performed by densitometric analysis (TotalLab; Nonlinear Dynamics, Newcastle on Tyne, UK).

### Real time PCR

Total RNA was obtained using TriPure isolation reagent (Roche Applied Science, Indianapolis, IN) following the manufacturer's instructions. RNA concentration was determined fluorometrically using RiboGreen reagent (Invitrogen, Carlsbad, CA). RNA integrity was checked by electrophoresis on 1.2% agarose gel containing 0.02 mol/L morpholinopropanesulfonic acid and 18% formaldehyde. Total mRNA was retrotranscribed using an iScript cDNA synthesis kit (Bio-Rad Laboratories). Transcript levels were determined by real-time PCR using the SsoFast EvaGreen supermix and the MiniOpticon thermal cycler (Bio-Rad Laboratories), normalizing the expression for TBP. Primer sequences (Invitrogen, Carlsbad, CA) were as follows:

VDR FW CCTCATAAAGTTCCAGGTGGGG

VDR RV GGATAGGCGGTCCTGAATGG

TBP FW TGTCCAGAGCACCAACAGTC

TBP RV TAACAGCAGCAAAACGCTTG

### Promoter analysis and chromatin immunoprecipitation assay (ChIP)

The promoter region (-6000bp, +500bp) of murine myogenin gene was analyzed for the presence of putative VDR binding sites (Matrix-Derived JASPAR CORE Models, Vertebrates, ID: MA0074.1) using the LASAGNA-Search web tool [55].

Chromatin was isolated from C2C12 myoblast untreated or treated during 4 hours with VitD in DMSO at a final concentration of 100 nM. ChIP was performed according to [56]. Cells were cross-linked adding to tissue culture medium PBS and 1% formaldehyde and incubating for 10 minutes. Crosslinking was blocked with 0.125 M glycine for 5 minutes. Cells were scraped and subsequently lysed in RIPA buffer (see above). Nuclei were pelleted by microcentrifugation and lysed by incubation in nuclear lysis buffer (1% sodium dodecyl sulfate, 10 mM EDTA, 50 mM Tris-chloride pH 8.1, 0.5 mM phenylmethylsulfonyl fluoride, protease inhibitors). The resulting chromatin solution was sonicated to obtain fragments of approximately 0.5 kb and immunoprecipitated with 3.5 μg of rabbit IgG or VDR (rabbit polyclonal antibody, clone C-20; Santa Cruz Biotechnology, Santa Cruz, CA). Immunoprecipitation procedures were performed according to [57]. Quantitative Real Time PCR was performed using SYBR green IQ reagent (Bio-Rad Laboratories) with CFX Connect detection system (Bio-Rad Laboratories). Primer sequences:

β-globin

FW GACAAACATTATTCAGAGGGAGT

RV AAGCAAATGTGAGGAGCAACTGAT

Cytochrome c

FW GGAGGCAAGCATAAGACTGG

RV TCCATCAGGGTATCCTCTCC

Probe 1

FW TGCAATGTCACAGAGGTCTAAGC

RV CTACACAGAAGGAGACAGAGGCTC

Probe 2

FW CTCCCTCTTCCTTCTCCTTCAGC

RV CAACCCAGGTCAGCCTATCAGTA

Probe 3

FW CACACAGGGTAGCAGGTAGATGAC

RV CACTATCATACCTTGCTTACCCAC

Probe 4

FW CAGGAACCTGCAAGGCATCAAAG

RV GTCATACCTGCTGTGGAAACTGC

Probe 5

FW AAGGAGGAAACAGGTGTGTGAGG

RV GCTGCACTTTGTATCCCC

Probe 6

FW CGTTCAACACACTTTCCACCTCC

RV GCAGTTTCCACAGCAGGTATGAC

Probe 7

FW TAGTCGGCCATCATTGGGAAGAG

RV CCTACCCACTCATTCCCACTTCT

### Immunofluorescence

C2C12 or primary cells were washed with PBS and fixed in 1:1 acetone:methanol. Samples were then probed with the following primary antibodies: MyHC (mouse monoclonal antibody, clone MY32), VDR (mouse monoclonal antibody, clone D6. Santa Cruz Biotechnology, Santa Cruz, CA), Myogenin (mouse monoclonal antibody, clone F5D. Santa Cruz Biotechnology, Santa Cruz, CA). Detection was performed using Alexa Fluor secondary antibodies (Life Technologies, Grand Island, NY). Nuclei were stained with DAPI fluorochrome, and the images were captured using an epi-illuminated fluorescence microscope (Axiovert 35; Carl Zeiss MicroImaging GmbH, Jena, Germany). About 200 myotubes/conditions were analyzed to assess the number of nuclei and myotube diameter.

### Statistical analysis

All the results are expressed as means ± SD, except for Real Time PCR gene expression (means ± SEM). Representative Western blots show independent samples. The significance of the differences was evaluated by ANOVA followed by Tukey's test.

## SUPPLEMENTARY MATERIALS FIGURES AND TABLES


