# Supplementary material for: Vitamin D and VDR in cancer cachexia and muscle regeneration
Source: Oncotarget. 2017 Feb 21;8(13):21778–93. doi: 10.18632/oncotarget.15583 (PMC5400623; doi:10.18632/oncotarget.15583)
Supplement: Supplementary file 1 [file oncotarget-08-21778-s001.pdf]

Vitamin D and VDR in cancer cachexia and muscle regeneration

SUPPLEMENTARY FIGURES

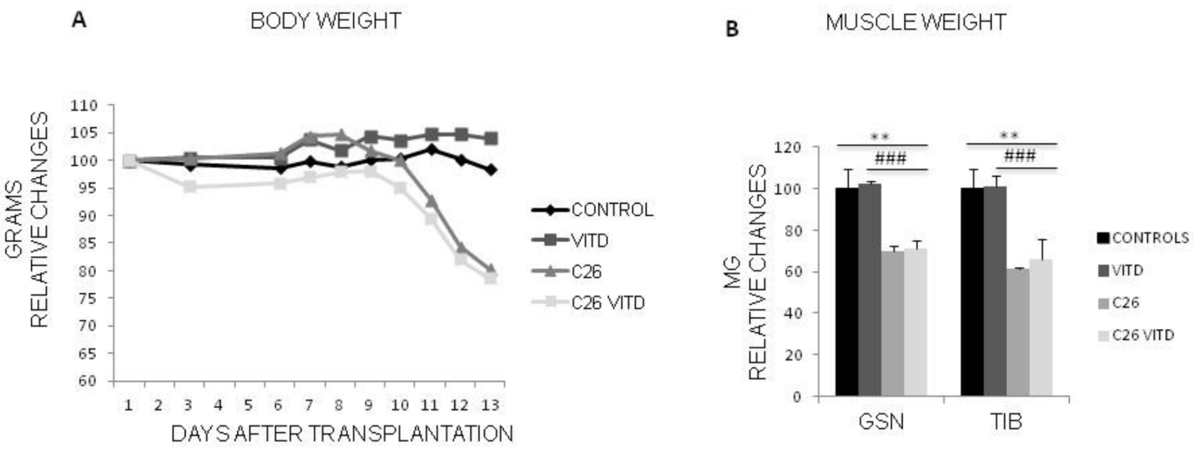

Supplementary Figure 1: Effects of VitD3 administration to mice bearing the C26 tumor. A. Body weight changes; **B.** gastrocnemius and tibialis muscle weight in controls and tumor-bearing mice either untreated or receiving VitD3. Data (means±SD) are expressed as percentages of controls (C, VIT D: n=6, C26, C26 VIT D: n=8). Significance of the differences: *p< 0.01 *vs* control; #p< 0.001 *vs* VIT D.

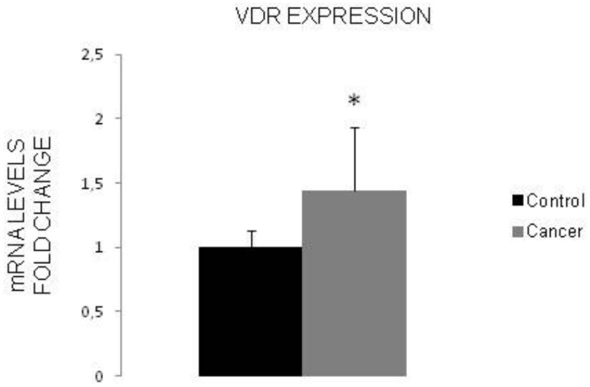

Supplementary Figure 2: VDR mRNA levels in the skeletal muscle of cancer patients. VDR mRNA levels have been evaluated by qPCR in the *rectus abdominis* muscle of controls (n = 4) and cancer patients (n = 14). For experimental details see Materials and Methods. Data (means±SEM) are expressed as fold change relative to control levels. Significance of the differences: * p < 0.05 vs controls.

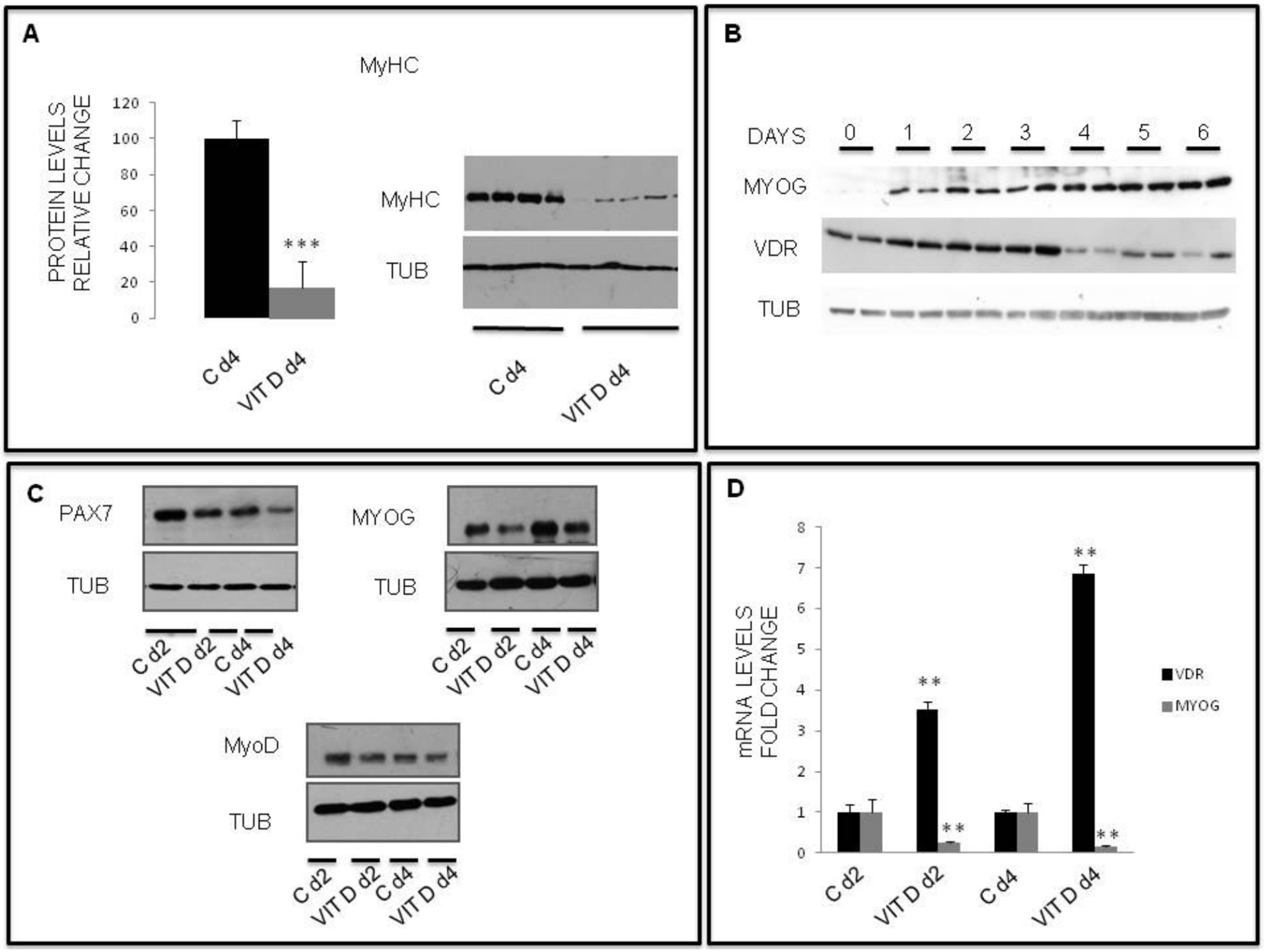

Supplementary Figure 3: Effects of VitD on C2C12 myoblast differentiation. A. MyHC protein levels in control or VitD-stimulated C2C12 cells after 4 days of differentiation (Sigma: anti MyHC antibody, clone MY32). Data (means ± SD) are expressed as % of controls. Significance of the differences: ***p<0.001*vs* C; **B.** western blotting representative images of VDR and myogenin protein levels in differetiating C2C12 cells (Santa Cruz Biotechnology: anti-VDR antibody, clone D6; anti-myogenin antibody, clone F5D); **C.** western blotting representative images of some myogenic regulatory factors expressed in control or VitD-stimulated C2C12 cells at day 2 and 4 of differentiation (quantification reported in Figure 3C; Santa Cruz Biotechnology: anti-myogenin antibody, clone F5D, anti-MyoD antibody, clone M318; Developmental Studies Hybridoma Bank, University of Iowa: anti-Pax7 antibody); **D.** mRNA levels of VDR and myogenin in control or VitD-stimulated C2C12 cells at day 2 and 4 of differentiation. Data (means±SEM) are expressed as fold change (C d2 and C d4 = 1). Significance of the differences: **p< 0.01 *vs* control.

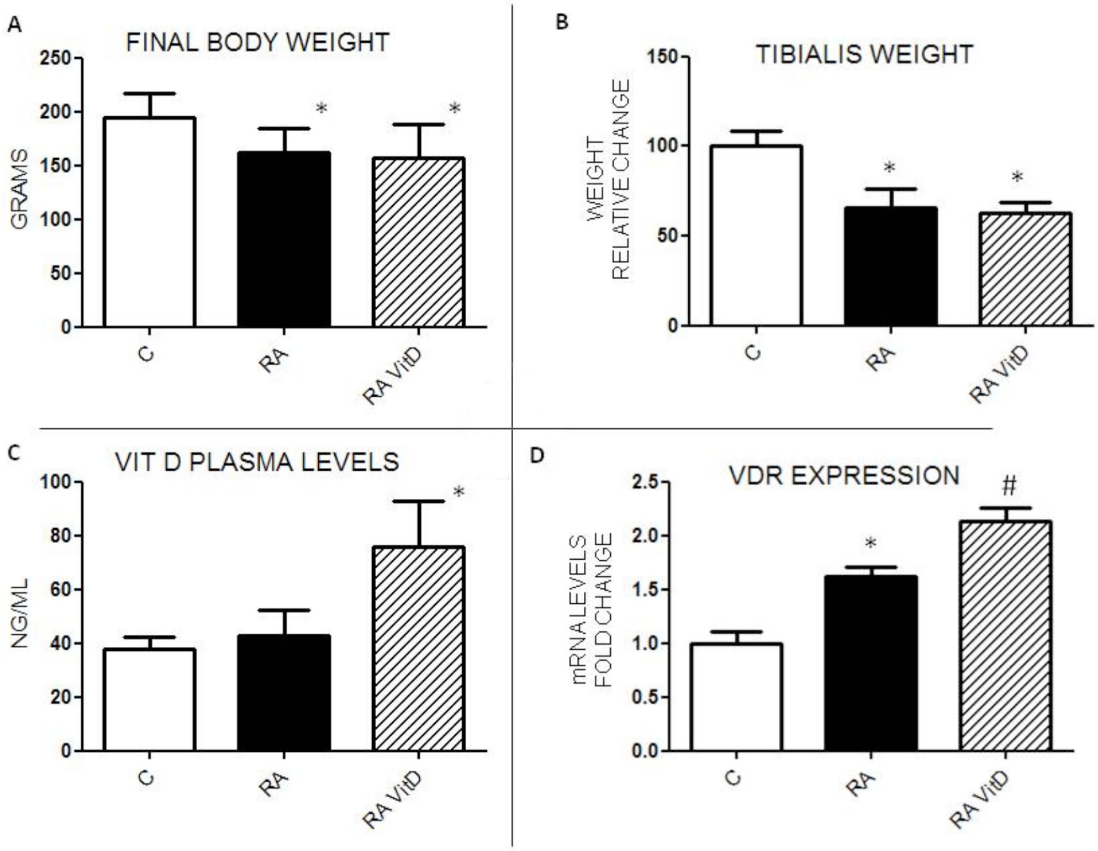

Supplementary Figure 4: Effects of VitD3 administration in rats affected by experimental rheumatoid arthritis. A. Body weight changes and **B.** tibialis muscle weight in control and RA rats either untreated or receiving VitD. **C.** VitD plasma levels as measured at the end of the experiment. **D.** VDR mRNA expression levels in the tibialis muscle. Data (means±SEM) are expressed as percentages of controls (n=6). Significance of the differences: *$p < 0.05$ *vs* C; #$p < 0.05$ *vs* RA.
